# National, Regional, State, and Selected Local Area Vaccination Coverage Among Adolescents Aged 13–17 Years — United States, 2013

**Published:** 2014-07-25

**Authors:** Laurie D. Elam-Evans, David Yankey, Jenny Jeyarajah, James A. Singleton, C. Robinette Curtis, Jessica MacNeil, Susan Hariri

**Affiliations:** 1 Immunization Services Division, National Center for Immunization and Respiratory Diseases; 2 Division of Bacterial Diseases, National Center for Immunization and Respiratory Diseases; 3 Division of Sexually Transmitted Diseases, National Center for HIV/AIDS, Viral Hepatitis, STD, and TB Prevention, CDC

The Advisory Committee on Immunization Practices (ACIP) recommends that adolescents routinely receive 1 dose of tetanus toxoid, reduced diphtheria toxoid, and acellular pertussis (Tdap) vaccine, 2 doses of meningococcal conjugate (MenACWY) vaccine, and 3 doses of human papillomavirus (HPV) vaccine (1,2).* ACIP also recommends administration of “catch-up”† vaccinations, such as measles, mumps, and rubella (MMR), hepatitis B, and varicella, and, for all persons aged ≥6 months, an annual influenza vaccination (1). ACIP recommends administration of all age-appropriate vaccines during a single visit (3). To assess vaccination coverage among adolescents aged 13–17 years, CDC analyzed data from the 2013 National Immunization Survey-Teen (NIS-Teen).§ This report summarizes the results of that analysis, which show that from 2012 to 2013, coverage increased for each of the vaccines routinely recommended for adolescents: from 84.6% to 86.0% for ≥1 Tdap dose; from 74.0% to 77.8% for ≥1 MenACWY dose; from 53.8% to 57.3% for ≥1 HPV dose among females, and from 20.8% to 34.6% for ≥1 HPV dose among males. Coverage varied by state and local jurisdictions and by U.S. Department of Health and Human Services (HHS) region. *Healthy People 2020* vaccination targets for adolescents aged 13–15 years (4) were reached in 42 states for ≥1 Tdap dose, 18 for ≥1 MenACWY dose, and 11 for ≥2 varicella doses. No state met the target for ≥3 HPV doses.¶ Use of patient reminder and recall systems, immunization information systems, coverage assessment and feedback to clinicians, clinician reminders, standing orders, and other interventions can help make use of every health care visit to ensure that adolescents are fully protected from vaccine-preventable infections and cancers (5), especially when such interventions are coupled with clinicians’ vaccination recommendations.

Vaccination coverage was assessed using 2013 NIS-Teen data for adolescents aged 13–17 years in the 50 states, the District of Columbia, selected local areas,** Guam, and the U.S. Virgin Islands, using a random-digit–dialed sample of landline and cell phones.†† Telephone interviews were conducted with the parent or guardian of age-eligible adolescents to obtain information about the adolescent’s demographic characteristics and to request vaccination provider contact information.§§ After receiving a respondent’s consent, a questionnaire was mailed to each vaccination provider to obtain provider-confirmed immunization information. In 2013, national estimates were based on responses for 18,264 adolescents (8,710 females and 9,554 males).¶¶ Details of NIS-Teen methodology, including methods for synthesizing provider-reported immunization histories and weighting, have been described previously.*** NIS-Teen data from 2006–2013 were used in this report to describe vaccination coverage over time. Weighted linear regression††† was used to assess coverage trends for vaccines recommended routinely for adolescents. T-tests were used to assess vaccination coverage differences by survey year (2013 compared with 2012), age, sex, race/ethnicity, and poverty status for all vaccines included in this report. Results were considered statistically significant at p<0.05.

## National Vaccination Coverage

During 2006–2013, NIS-Teen data show that coverage trends differed substantially for Tdap, MenACWY, and HPV vaccination (Figure). Coverage estimates for ≥1 Tdap dose and ≥1 MenACWY dose increased significantly each year from 2006 to 2013, with average increases of 10.4 percentage points (95% confidence interval [CI] = 7.8–13.1) for Tdap and 8.9 percentage points (CI = 6.5–11.3) for MenACWY. Coverage for ≥1 HPV dose increased an average of 4.5 percentage points (CI = 2.7–6.3) annually from 2007 to 2013 for females, and by 9.9 percentage points (CI = 4.8–15.0) from 2010 to 2013 for males. In 2013, Tdap and MenACWY coverage estimates were 86.0% and 77.8%, respectively (Table 1). From 2012 to 2013, coverage with ≥1, ≥2, and ≥3 HPV doses increased for both sexes. Coverage with ≥1 HPV dose in 2013 was 57.3% for females and 34.6% for males. No statistically significant changes occurred from 2012 to 2013 in coverage for ≥2 doses of MMR vaccine or ≥3 doses of hepatitis B vaccine. However, coverage for ≥2 doses of varicella vaccine increased from 74.9% to 78.5% among adolescents with no history of disease (Table 1).

Coverage with the second MenACWY dose was calculated as the proportion of adolescents aged 17 years on date of interview who received a second MenACWY dose on or after their 16th birthday, among those who had received a first dose before their 16th birthday (only second doses received on or after their 16th birthday and at least 8 weeks after the first dose were counted). All of these adolescents were aged 16 years after the MenACWY second dose was recommended by ACIP in October 2010 (n = 2,310) (6). The MenACWY 2-dose completion rate was 29.6% (CI = 26.4%–33.0%).

## Vaccination Coverage by Selected Characteristics

In 2013, among females, ≥1 HPV dose coverage was significantly higher among adolescents aged 15–17 years compared with younger adolescents (Table 1). However, ≥1 HPV dose coverage for males did not vary by age. In 2013, as found previously, most vaccination coverage rates were similar by sex; however, females had greater vaccination coverage than males for ≥1, ≥2, and ≥3 HPV doses and 3-dose HPV series completion§§§ (Table 1). Also, females had significantly higher vaccination coverage than males for ≥2 varicella doses (80.0% [CI = 78.1%–81.7%] versus 77.2% [CI = 75.2%–79.0%]).

In 2013, there were no racial or ethnic differences in vaccination coverage for ≥1 Tdap, ≥3 hepatitis B, or ≥2 varicella (Table 2). However, ≥1 MenACWY dose coverage was higher among Hispanic and Asian adolescents compared with white adolescents. Among females, ≥1, ≥2, and ≥3 HPV dose coverage was higher among Hispanic compared with white adolescents. Among males, ≥1, ≥2, and ≥3 HPV dose coverage was higher among black and Hispanic adolescents compared with white adolescents. Black adolescent females had lower HPV 3-dose series completion compared with white adolescent females and, in contrast to findings in 2012, series completion among Hispanic females was similar to coverage among white adolescent females. There were no statistically significant racial/ethnic differences among males for HPV 3-dose series completion. In 2013, vaccination coverage did not vary by poverty level¶¶¶ for ≥1 Tdap, ≥1 MenACWY, ≥2 MMR, ≥ 3 hepatitis B, ≥2 varicella, or HPV 3-dose series completion (for males or females) (Table 2). However, those living below the poverty level had higher ≥1, ≥2, and ≥3 HPV dose coverage (for males) and ≥1 and ≥2 HPV dose coverage (for females), compared with their counterparts living at or above the poverty level. These findings in 2013 data that females had no difference in 3-dose HPV completion by poverty status were not observed in 2012 (7).

## State and Regional Vaccination Coverage

In 2013, there was wide variation among states in coverage (Table 3). Coverage for ≥1 Tdap ranged from 60.2% (Mississippi) to 95.5% (Rhode Island), whereas coverage estimates for ≥1 MenACWY ranged from 40.4% (Arkansas) to 93.7% (North Dakota). Among females, coverage for ≥1 HPV doses ranged from 39.9% (Kansas) to 76.6% (Rhode Island) and for ≥3 HPV doses ranged from 20.5% (Utah) to 56.5% (Rhode Island). For males, coverage for ≥1 HPV doses ranged from 11.0% (Utah) to 69.3% (Rhode Island) and for ≥3 HPV doses ranged from 7.3% (Nevada) to 43.2% (Rhode Island). Coverage for ≥2 MMR doses ranged from 83.2% (West Virginia) to 97.4% (New Hampshire and Louisiana). Coverage for ≥2 varicella doses ranged from 50.6% (South Dakota) to 95.8% (Connecticut).

Coverage with ≥1 HPV doses in females increased from 2012 to 2013 in five states (Illinois, Michigan, New Hampshire, New Mexico, and South Carolina), with percentage point increases ranging from 12.0 (Illinois) to 18.5 (South Carolina). HPV coverage with ≥1 doses in females also increased by 6.0 percentage points (CI = 0.1–12.0) in HHS Region IV (southeastern states) and by 7.8 percentage points (CI = 2.1–13.4) in HHS Region V (north central states) (Table 3).

## *Healthy People 2020* Targets

The *Healthy People 2020* national targets for vaccination coverage among adolescents aged 13–15 years are 80.0% for ≥1 Tdap dose, ≥1 MenACWY dose, and ≥3 HPV doses (among females) and 90.0% for ≥2 varicella doses (4). Among adolescents aged 13–15 years, vaccination coverage in 2013 was 87.5% (CI = 86.4%–88.6%) for ≥1 Tdap dose, 78.1% (CI = 76.7%–79.4%) for ≥1 MenACWY dose, 32.7% (CI = 30.3%–35.2%) for ≥3 HPV doses (among females), and 80.7% (79.2%–82.1%) for ≥2 varicella doses. From 2012 to 2013, vaccination coverage for these national targets increased by 2.2–4.6 percentage points. The number of states meeting or exceeding the target was 42 for ≥1 Tdap dose (up from 36 in 2012), 18 for ≥1 MenACWY dose (up from 12 in 2012), 11 for ≥2 varicella doses (up from 9 in 2012), and for ≥3 HPV doses among females, none.

### Discussion

From 2012 to 2013, coverage for adolescents aged 13–17 years increased for all vaccinations routinely recommended for adolescents, with increases ranging from 1.4 percentage points for ≥1 Tdap dose to 13.8 percentage points for ≥1 HPV dose in males. Nationally, the *Healthy People 2020* vaccination coverage target for adolescents aged 13–15 years was reached for Tdap (87.5%) for the third survey year, and progress continues for MenACWY (78.1%) and varicella (80.7%). These high vaccination coverage levels confirm that established targets of 80%–90% are achievable for adolescents for vaccination and vaccination series, just as they are for young children. However, coverage for ≥3 HPV doses among females aged 13–15 years in 2013 was 32.7%, and trends measured by 2013 and earlier NIS-Teen data demonstrate that the 80% *Healthy People 2020* target will be difficult to achieve without changes in clinical practices, leaving adolescents vulnerable to develop the cancers that safe, effective HPV vaccines can prevent. Accelerating progress in HPV vaccination will require the collaboration of numerous stakeholders (e.g., clinicians, parents, adolescents, and public health professionals) to overcome barriers to use of HPV vaccines (8). A variety of factors, including knowledge, attitudes, and behaviors among clinicians and parents likely contribute to lower HPV vaccination initiation compared with Tdap and MenACWY vaccinations. Addressing barriers to HPV vaccination at the recommended ages of 11–12 years could reduce missed opportunities to administer all recommended adolescent vaccines during the same clinical encounter. Another analysis of 2013 NIS-Teen data indicates that for adolescent females born in 2000, coverage with at least 1 dose of HPV vaccine before age 13 years could have reached 91.3% if opportunities to administer HPV vaccine when other vaccines were given had not been missed (9).

Although HPV vaccination of adolescent females increased by only 3.5 percentage points from 2012 to 2013, this increase was significantly greater than that observed from 2011 to 2012, when first dose HPV coverage among adolescent females stagnated. Whether increased health promotion activities aimed at clinicians (e.g., http://www.cdc.gov/vaccines/youarethekey) and parents initiated during 2013 account for the modest increase is not known. Vaccination coverage increases in 2013 were primarily observed in the last quarter of the year, which could reflect the impact of health promotion activities initiated during the summer and fall of 2013.

The high number of measles cases reported in the United States in 2014 (580 cases through July 18) (http://www.cdc.gov/measles/index.html) is a reminder of the importance of achieving and maintaining high 2-dose MMR vaccination coverage among children and adolescents throughout the country. Whereas eight states had 2-dose coverage >95%, 13 states and the District of Columbia had 2-dose coverage <90%, reflecting a vulnerability to measles transmission.

In 2013, there were racial and ethnic differences for some vaccines (MenACWY, MMR, and HPV). Compared with whites, vaccination coverage among Hispanics was higher for ≥1 MenACWY dose and each HPV dose among males and females, but lower for ≥2 MMR doses. Vaccination coverage was similar by poverty level except for HPV vaccination, with higher coverage with ≥1, ≥2, and ≥3 HPV doses for males and ≥1 and ≥2 HPV doses for females among those living below poverty level compared with those living at or above the poverty level. The higher coverage among some racial/ethnic minorities and those living below poverty level might be partly attributable to the continued effectiveness of the Vaccines for Children program (VFC), which provides recommended vaccines at no cost to eligible children.**** However, the significantly lower rates of HPV vaccine series completion in black females compared with white females warrants investigation of possible differences (e.g., access to quality care, such as access to clinicians with reminder-recall systems) that might limit vaccine series completion in some populations. Learning what factors are fostering achievement of increasing and comparatively higher HPV vaccination coverage among Hispanic adolescents might inform strategies for the general population. The similar or higher vaccination coverage among adolescents living below the poverty threshold contrasts with findings for coverage with some early childhood vaccinations (10). Among children aged 19–35 months, poverty has been associated with lower coverage of newer vaccines (e.g., rotavirus), and some vaccines that require doses during the second year of life (e.g., DTaP and PCV) (10).

What is already known on this topic?

The Advisory Committee on Immunization Practices (ACIP) recommends that adolescents receive 1 dose of tetanus toxoid, reduced diphtheria toxoid and acellular pertussis (Tdap) vaccine, 2 doses of meningococcal conjugate (MenACWY) vaccine, and 3 doses of human papillomavirus (HPV) vaccine. ACIP also recommends administration of these and all age-appropriate vaccines during a single visit. During 2006–2012, national vaccination coverage for ≥1 Tdap and ≥1 MenACWY increased steadily, with Tdap coverage in 2011 reaching national target levels for adolescents. During 2007–2011, coverage for ≥1 HPV vaccine dose among females increased steadily, but from 2011 to 2012, there were no changes in coverage. Coverage for ≥1 HPV vaccine dose among males increased from 2011–2012.

What is added by this report?

From 2012 to 2013, vaccination coverage among U.S. adolescents increased to 86.0% for ≥1 dose of Tdap vaccine, 77.8% for ≥1 dose of MenACWY vaccine, 57.3% for ≥1 dose of HPV vaccine among females, and 34.6% for ≥1 dose of HPV vaccine among males. Vaccination coverage levels continued to vary widely among states. Although HPV vaccination coverage increased among both females and males, levels are still low and reflect many missed opportunities. Five states had substantial increases in HPV coverage from 2012 to 2013, suggesting greater progress is feasible.

What are the implications for public health practice?

Lower vaccination coverage for HPV compared with Tdap and MenACWY vaccines indicates clinicians, parents, and adolescents are missing opportunities for infection and cancer prevention. Clinician recommendations strongly influence the decisions of parents to vaccinate their children; to maximize coverage, clinicians should clearly and consistently recommend all ACIP-recommended vaccines, including HPV. Health care systems interventions, including use of client reminder and recall systems, immunization information systems, clinician reminders, and standing orders, should be employed to improve protection of adolescents from vaccine-preventable infections and future cancers.

Geographic differences in coverage continue to vary by vaccine. Factors contributing to state or regional differences might include different state school vaccination requirements, different stages of vaccine policy implementation, increased vaccine demand in response to local disease, differing parental knowledge and attitudes toward or access to vaccination, inconsistent clinician adherence to and knowledge about vaccine recommendations, and other factors. Although there was an overall increase in HPV vaccination coverage among females, there was continued wide variability among states and HHS Regions. HPV coverage among females increased significantly from 2012 to 2013 in only five states (Illinois, Michigan, New Hampshire, New Mexico, and South Carolina) for ≥1 HPV dose and in four states (Illinois, Mississippi, New Mexico, and South Carolina) for ≥3 HPV doses. These states have undertaken diverse initiatives that likely contributed to the significant increases in HPV vaccination coverage, including 1) developing partnerships with state chapters of the American Academy of Pediatrics and with the Academy of Family Physicians to promote HPV immunization, 2) working actively with Immunization Coalitions and Cancer Collaboratives to incorporate HPV immunization into strategic plans and ensuring that clinical and immunization conferences highlight HPV vaccination topics, 3) developing an HPV Vaccine Task Force to discuss and facilitate HPV vaccination health promotion activities and interventions, 4) providing peer-to-peer physician HPV vaccination training onsite, and 5) increasing provider assessment and feedback visits focused on increasing vaccination coverage among adolescents. Understanding the extent to which vaccination programs and policies, provider practices, and parental knowledge and access influence these geographic differences might help inform public health action.

The findings in this report are subject to at least three limitations. First, the household response rates for landline and cell phone samples were 51.1% and 23.3%, respectively. Furthermore, only 59.5% of landline and 54.5% of cell phone completed interviews had adequate vaccine provider data. Therefore, estimates might have been biased, even after weighting adjustments for nonresponse and exclusion of households without telephones. A total survey error model of 2011 NIS-Teen that included comparison with provider-reported data collected from a sample of National Health Interview Survey participants indicated coverage estimates were approximately 2, 3, and 6 percentage points too high for Tdap, MenACWY, and HPV (among females) vaccinations, respectively, as a result of noncoverage and nonresponse error.†††† Estimates of bias do not include errors in vaccination status (e.g., underascertainment from incomplete vaccination provider identification and unknown medical record completeness) (7). Second, although response rates have been stable in recent years and weights have been adjusted to reflect the increasing prevalence of cell phone–only households over time, it is possible that nonresponse bias might have changed over time, which could affect interpretation of comparisons across data years. Finally, some of the state-specific and racial/ethnic-specific analyses might be unreliable because of small sample sizes (7). Estimates with confidence half-widths wider than 10 are less reliable, and this impacts estimates for some racial and ethnic populations. For HPV coverage analyses by state and sex, small sample sizes decrease the power to detect differences.

High Tdap coverage levels among adolescents aged 13–17 years indicate that similar coverage levels are attainable for other vaccines recommended for adolescents. Improved adherence of clinicians and parents to the ACIP recommendation to administer all age-appropriate vaccines during a single visit could substantially increase lagging vaccination coverage levels. At each encounter with a clinician, every adolescent’s immunization history should be reviewed to ensure complete vaccination consistent with ACIP recommendations. Additionally, clinicians should provide strong, consistent recommendations for all ACIP-recommended vaccines. HPV vaccine should be recommended with the same emphasis and at the same time as the other vaccines for adolescents. Recommended strategies to improve vaccination coverage include use of combinations of strategies such as patient reminder and recall systems, standing orders, and use of immunization information systems (5). Coverage levels should continue to be monitored to describe coverage disparities, to use estimates to identify target populations for interventions to increase coverage, and to inform development of additional policies that will support further efforts to reduce vaccine-preventable diseases, including cancers.

## Figures and Tables

**FIGURE:**
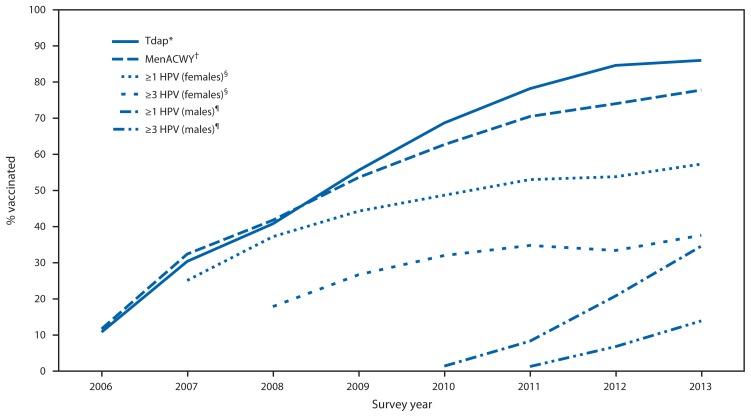
Estimated vaccination coverage with selected vaccines and doses among adolescents aged 13–17 years, by survey year — National Immunization Survey-Teen, United States, 2006–2013 **Abbreviations:** Tdap = tetanus toxoid, reduced diphtheria toxoid, and acellular pertussis; MenACWY = meningococcal conjugate; HPV = human papillomavirus. * ≥1 dose Tdap vaccine on or after age 10 years. ^†^ ≥1 dose MenACWY vaccine. ^§^ HPV vaccine, either bivalent or quadrivalent, among females. The Advisory Committee on Immunization Practices (ACIP) recommends either bivalent or quadrivalent vaccine for females. ^¶^ HPV vaccine, either bivalent or quadrivalent, among males. ACIP recommends the quadrivalent vaccine for males; however, some males might have received bivalent vaccine.

**TABLE 1:** Estimated vaccination coverage with selected vaccines among adolescents aged 13–17 years,* by age at interview — National Immunization Survey–Teen (NIS-Teen), United States, 2013

	Age at interview (yrs)										Total			
		
	13 (n = 3,735)		14 (n = 3,841)		15 (n = 3,645)		16 (n = 3,783)		17 (n = 3,260)		2013 (N = 18,264)		2012† (N = 19,199)	
							
Vaccine	%	(95% CI)	%	(95% CI)	%	(95% CI)	%	(95% CI)	%	(95% CI)	%	(95% CI)	%	(95% CI)
Tdap§ ≥ 1 dose	87.2	(±1.9)	87.0	(±2.1)	88.4	(±1.7)	84.3	(±2.1)	83.0	(±2.7)¶	86.0	(±0.9)**	84.6	(±0.9)
MenACWY†† ≥1 dose	76.1	(±2.4)	78.2	(±2.3)	80.0	(±2.3)¶	77.8	(±2.5)	76.7	(±2.9)	77.8	(±1.1)**	74.0	(±1.1)
HPV§§ vaccination														
Females														
≥1 dose	50.6	(±4.1)	55.1	(±4.2)	58.8	(±4.3)¶	60.0	(±4.5)¶	62.3	(±4.5)¶	57.3	(±1.9)**	53.8	(±1.9)
≥2 dose	39.2	(±4.2)	43.3	(±4.2)	48.7	(±4.5)¶	51.1	(±4.6)¶	56.8	(±4.5)¶	47.7	(±2.0)**	43.4	(±1.9)
≥3 doses	25.8	(±3.8)	32.1	(±3.9)¶	39.4	(±4.6)¶	43.1	(±4.5)¶	48.2	(±4.5)¶	37.6	(±1.9)**	33.4	(±1.7)
Males														
≥1 dose	33.5	(±4.5)	35.1	(±4.4)	36.2	(±4.1)	35.9	(±4.0)	32.1	(±4.1)	34.6	(±1.9)**	20.8	(±1.5)
≥2 dose	23.4	(±4.3)	24.3	(±4.0)	23.8	(±3.8)	23.2	(±3.7)	22.9	(±3.5)	23.5	(±1.7)**	12.7	(±1.3)
≥3 doses	11.7	(±2.7)	13.6	(±3.3)	15.3	(±3.5)	13.7	(±3.1)	15.1	(±3.0)	13.9	(±1.4)**	6.8	(±1.0)
HPV§§ 3-dose series completion¶¶														
emsp;Females	56.1	(±6.7)	64.7	(±5.7)	72.1	(±5.0)¶	75.9	(±5.6)¶	79.5	(±4.6)¶	70.4	(±2.5)**	66.7	(±2.6)
Males	41.6	(±9.4)	47.1	(±9.3)	51.0	(±8.7)	48.8	(±8.2)	53.4	(±8.5)	48.3	(±4.0)	45.1	(±5.0)
MMR*** ≥2 doses	92.6	(±1.4)	93.1	(±1.4)	91.4	(±2.1)	92.0	(±1.6)	89.7	(±2.3)¶	91.8	(±0.8)	91.4	(±0.8)
Hepatitis B ≥3 doses	94.7	(±1.3)	94.0	(±1.3)	92.5	(±1.9)	93.1	(±1.5)	91.4	(±2.2)¶	93.2	(±0.7)	92.8	(±0.7)
Varicella														
History of varicella†††	15.6	(±2.1)	19.5	(±2.4)¶	25.1	(±2.5)¶	30.6	(±2.8)¶	37.1	(±3.0)¶	25.4	(±1.2)**	30.6	(±1.2)
Among adolescents with no history of varicella														
≥1 dose vaccine	97.4	(±0.8)	95.4	(±1.6)¶	94.6	(±2.0)¶	94.0	(±1.9)¶	91.9	(±3.3)¶	94.9	(±0.9)	94.7	(±0.8)
≥2 doses vaccine	83.1	(±2.2)	80.2	(±2.5)	78.7	(±3.0)¶	76.6	(±3.1)¶	71.6	(±4.0)¶	78.5	(±1.3)**	74.9	(±1.4)
History of varicella or received ≥2 doses varicella vaccination	85.7	(±1.9)	84.1	(±2.1)	84.0	(±2.3)	83.7	(±2.3)	82.2	(±2.8)¶	84.0	(±1.0)	82.6	(±1.0)

**Abbreviations:** CI = confidence interval; Tdap = tetanus toxoid, reduced diphtheria toxoid, and acellular pertussis; MenACWY = meningococcal conjugate; HPV = human papillomavirus; MMR = measles, mumps, and rubella.

* Adolescents (N = 18,264) in the 2013 NIS-Teen were born January 11, 1995, through February 13, 2001.

† Estimates for overall NIS-Teen data for 2012 are provided as a comparison with overall 2013 NIS-Teen data.

§ Includes percentages receiving Tdap vaccine at or after age 10 years.

¶ Statistically significant difference (p<0.05) in estimated vaccination coverage by age: reference group was adolescents aged 13 years.

** Statistically significant difference (p<0.05) compared with 2012 NIS-Teen overall estimates.

†† Includes percentages receiving MenACWY or meningococcal-unknown type vaccine.

§§ HPV vaccine, either quadrivalent or bivalent may be used for females, and only quadrivalent may be used for males. Percentage reported among females (n = 8,710) and males (n = 9,554). Some adolescents might have received more than the recommended 3 doses of HPV vaccine.

¶¶ The completion rate for the 3-dose HPV vaccination series represents the percentage of adolescents who received ≥3 doses among those who had ≥1 HPV vaccine dose with at least 24 weeks between the first dose and the interview date. The calculation was limited to 4,611 females and 2,580 males who met the criteria of having received ≥1 HPV vaccine dose and having at least 24 weeks between the first dose and the interview date.

*** ≥2 doses of MMR vaccine.

††† By parent/guardian report or provider records.

**TABLE 2:** Estimated vaccination coverage among adolescents aged 13–17 years,* by race/ethnicity,† poverty level,§ and selected vaccines and doses — National Immunization Survey–Teen (NIS-Teen), United States, 2013

	Race/Ethnicity												Poverty status			
		
	White, non-Hispanic (n = 12,064)		Black, non-Hispanic (n = 1,647)		Hispanic (n = 2,741)		American Indian/Alaska Native, non-Hispanic (n = 284)		Asian, non-Hispanic (n = 561)		Multiracial (n = 886)		Below poverty level (n = 3,078)		At or above poverty level (n = 14,754)	
								
Vaccines	%	(95% CI)¶	%	(95% CI)	%	(95% CI)	%	(95% CI)	%	(95% CI)	%	(95% CI)	%	(95% CI)	%	(95% CI)
Tdap** ≥1 dose	85.9	(±1.1)	84.1	(±3.0)	87.1	(±2.4)	85.3	(±7.2)	89.7	(±3.6)	85.4	(±4.9)	85.2	(±2.3)	86.4	(±1.0)
MenACWY †† ≥1 dose	75.6	(±1.3)	77.0	(±3.3)	83.4	(±2.8)§§	71.7	(±11.1)	83.8	(±7.1)§§	76.3	(±5.1)	78.4	(±2.6)	77.5	(±1.2)
HPV¶¶ vaccination																
Females																
≥1 dose	53.1	(±2.3)	55.8	(±5.2)	67.5	(±5.0)§§	73.3	(±14.7)§§	57.0	(±11.4)	57.6	(±9.3)	66.8	(±4.3)§§	54.6	(±2.2)
≥2 dose	44.0	(±2.2)	45.6	(±5.2)	57.7	(±5.4)§§	57.3	(±15.2)	47.2	(±11.2)	46.2	(±9.5)	55.2	(±4.6)§§	45.3	(±2.2)
≥3 doses	34.9	(±2.1)	34.2	(±4.8)	44.8	(±5.6)§§	43.2	(±14.2)	40.4	(±11.0)	40.3	(±9.3)	41.5	(±4.6)	36.4	(±2.1)
Males																
≥1 dose	26.7	(±1.9)	42.2	(±5.5)§§	49.6	(±5.2)§§	38.6	(±14.0)	26.3	(±8.9)	34.5	(±7.3)§§	46.7	(±4.5)§§	30.8	(±2.0)
≥2 dose	18.5	(±1.7)	27.5	(±4.8)§§	34.5	(±5.3)§§	24.8	(±11.4)	19.5	(±8.0)	19.1	(±5.2)	28.7	(±4.0)§§	22.0	(±1.9)
≥3 doses	11.1	(±1.3)	15.7	(±3.8)§§	20.3	(±4.5)§§	NA	NA	9.1	(±4.5)	12.5	(±4.2)	16.7	(±3.0)§§	13.0	(±1.6)
HPV¶¶ 3-dose series completion***																
Females	71.8	(±2.9)	63.7	(±7.3)§§	69.5	(±6.1)	60.1	(±16.9)	77.2	(±12.1)	75.1	(±13.8)	66.2	(±5.7)	71.9	(±2.8)
Males	51.1	(±4.7)	44.8	(±8.8)	47.4	(±9.0)	48.4	(±20.0)	40.0	(±18.8)	49.3	(±13.9)	44.3	(±7.2)	50.4	(±4.8)
MMR††† ≥2 doses	92.8	(±0.8)	91.1	(±2.4)	90.2	(±2.3)§§	93.5	(±5.2)	90.8	(±6.0)	89.8	(±3.7)	91.7	(±1.7)	91.8	(±0.9)
Hepatitis B ≥3 doses	93.8	(±0.8)	93.2	(±2.1)	92.8	(±2.0)	93.4	(±5.3)	87.8	(±6.6)	91.7	(±3.1)	93.2	(±1.6)	93.1	(±0.9)
Varicella																
History of varicella§§§	26.8	(±1.4)	22.6	(±3.5)§§	24.6	(±3.0)	36.6	(±10.6)	24.2	(±6.7)	18.5	(±3.9)§§	29.0	(±3.0)§§	24.0	(±1.2)
Among adolescents with no history of varicella																
≥1 dose vaccine	95.3	(±0.8)	94.3	(±2.6)	94.5	(±2.5)	95.7	(±3.7)	94.3	(±6.7)	94.4	(±3.0)	94.7	(±1.9)	95.2	(±1.0)
≥2 dose vaccine	77.7	(±1.5)	77.9	(±3.6)	80.3	(±3.5)	78.7	(±9.8)	85.2	(±8.1)	76.7	(±6.4)	77.3	(±3.0)	79.0	(±1.5)
History of varicella or received ≥2 doses varicella vaccination	83.7	(±1.1)	82.9	(±3.0)	85.2	(±2.7)	86.5	(±6.4)	88.8	(±6.3)	81.0	(±5.4)	83.8	(±2.3)	84.0	(±1.1)

**Abbreviations:** CI = confidence interval; Tdap = tetanus toxoid, reduced diphtheria toxoid, and acellular pertussis; MenACWY = meningococcal conjugate; HPV = human papillomavirus; NA = not available (estimate not reported because unweighted sample size for the denominator was <30 or 95% CI half width/estimate >0.6); MMR = measles, mumps, and rubella.

* Adolescents (N = 18,264) in the 2013 NIS-Teen were born January 11, 1995, through February 13, 2001.

† Adolescent’s race/ethnicity was reported by parent or guardian. Adolescents identified in this report as white, black, Asian, American Indian/Alaska Native or multiracial were reported by the parent or guardian as non-Hispanic. Adolescents identified as multiracial had more than one race category selected. Adolescents identified as Hispanic might be of any race. Native Hawaiian or other Pacific Islanders were not included in the table because of small sample sizes.

§ Adolescents were classified as below poverty level if their total family income was less than the federal poverty level specified for the applicable family size and number of children aged <18 years. All others were classified as at or above the poverty level. Additional information available at http://www.census.gov/hhes/www/poverty.html. Poverty status was unknown for 432 adolescents.

¶ Estimates with 95% CI half-widths >10 might not be reliable.

** Includes percentages receiving Tdap vaccine at or after age 10 years.

†† Includes percentages receiving MenACWY and meningococcal-unknown type vaccine.

§§ Statistically significant difference (p<0.05) in estimated vaccination coverage by race/ethnicity or poverty level; referent groups were non-Hispanic white adolescents and adolescents living at or above poverty level, respectively.

¶¶ HPV vaccine, either quadrivalent or bivalent may be used for females, and only quadrivalent may be used for males. Percentage reported among females (n = 8,710) and males (n = 9,554). Some adolescents might have received more than the 3 recommended HPV vaccine doses.

*** The completion rate for the 3-dose HPV vaccination series represents the percentage of adolescents who received ≥3 doses among those who had ≥1 HPV vaccine dose with at least 24 weeks between the first dose and the interview date. The calculation was limited to 4,611 females and 2,580 males who met the criteria of having received ≥1 HPV vaccine dose and having ≥24 weeks between the first dose and the interview date.

††† Includes ≥2 doses of MMR vaccine.

§§§ By parent/guardian report or provider records.

**TABLE 3:** Estimated vaccination coverage with selected vaccines and doses* among adolescents aged 13–17 years† by HHS region and state/selected local area — National Immunization Survey–Teen (NIS-Teen), United States, 2013

Regional/State/Local area	≥2 MMR§	≥2 VAR¶	≥1 Tdap**	≥1 MenACWY††	Females (n = 8,264)			Males (n = 9,554)		
	
					≥1 HPV§§	≥2 HPV¶¶	≥3 HPV***	≥1 HPV§§	≥2 HPV¶¶	≥3 HPV***
									
	% (95% CI)†††	% (95% CI)	% (95% CI)	% (95% CI)	% (95% CI)	% (95% CI)	% (95% CI)	% (95% CI)	% (95% CI)	% (95% CI)
**United Sates overall**	**91.8 (±0.8)**	**78.5 (±1.3)** §§§	**86.0 (±0.9)** §§§	**77.8 (±1.1)** §§§	**57.3 (±1.9)** §§§	**47.7 (±2.0)** §§§	**37.6 (±1.9)** §§§	**34.6 (±1.9)** §§§	**23.5 (±1.7)** §§§	**13.9 (±1.4)** §§§
**HHS Region I**	**95.7 (±1.4)**	**90.9 (±2.3)**	**92.7 (±1.7)**	**87.7 (±2.1)**	**61.9 (±4.6)**	**51.8 (±4.8)**	**41.8 (±4.7)**	**51.4 (±4.6)** §§§	**36.9 (±4.5)** §§§	**23.0 (±3.9)** §§§
Connecticut	97.3 (±2.4)	95.8 (±3.2)	90.8 (±4.3)	90.6 (±4.2)	56.0 (±9.2)	49.0 (±9.3)	40.1 (±9.1)	52.3 (±9.2)§§§	36.4 (±8.9)§§§	23.4 (±7.9)§§§
Maine	88.8 (±4.4)¶¶¶	71.0 (±7.2)	83.0 (±4.7)	71.2 (±5.6)	60.2 (±8.8)	55.4 (±8.9)	45.8 (±8.8)	42.2 (±8.5)§§§	31.1 (±7.9)§§§	17.6 (±6.0)
Massachusetts	95.8 (±2.6)	91.1 (±4.2)	94.9 (±2.6)	89.6 (±3.6)	62.3 (±8.3)	48.9 (±8.6)	39.3 (±8.4)	52.8 (±8.2)§§§	37.8 (±8.0)§§§	21.8 (±7.0)
New Hampshire	97.4 (±2.2)	91.6 (±4.1)	94.7 (±2.9)	85.6 (±4.4)	68.0 (±8.3)§§§	57.2 (±8.6)§§§	43.2 (±8.6)	41.4 (±8.4)§§§	28.5 (±7.9)§§§	17.8 (±6.7)
Rhode Island	95.6 (±2.9)	93.2 (±3.6)	95.5 (±2.9)	92.0 (±3.5)	76.6 (±8.1)	68.5 (±8.7)	56.5 (±9.3)	69.3 (±8.5)§§§	58.0 (±9.0)§§§	43.2 (±9.0)§§§
Vermont	94.5 (±2.7)	90.9 (±4.5)	91.8 (±3.7)	79.2 (±5.3)	60.2 (±9.0)	53.5 (±9.2)	42.7 (±9.1)	41.3 (±8.8)§§§	26.3 (±8.0)	21.7 (±7.7)§§§
**HHS Region II**	**93.8 (±2.0)**	**79.1 (±3.6)**	**88.2 (±2.6)**	**86.1 (±2.6)**	**56.5 (±5.3)**	**50.2 (±5.4)**	**40.8 (±5.4)**	**36.6 (±5.1)** §§§	**28.1 (±4.8)** §§§	**17.5 (±4.1)** §§§
New Jersey	94.9 (±3.2)	79.2 (±6.6)	85.5 (±5.3)	91.8 (±4.1)	45.8 (±9.7)	39.1 (±9.6)	31.4 (±9.2)	32.4 (±8.9)§§§	25.7 (±8.4)§§§	14.2 (±7.0)
New York	93.3 (±2.5)	79.1 (±4.3)	89.5 (±2.9)	83.3 (±3.4)	61.7 (±6.2)	55.6 (±6.4)	45.4 (±6.6)	38.6 (±6.1)§§§	29.3 (±5.8)§§§	19.1 (±5.1)
NY-City of New York	90.9 (±4.2)	80.6 (±6.3)§§§	88.9 (±4.5)	83.0 (±5.2)	64.2 (±9.0)	56.1 (±9.4)	45.2 (±9.6)	46.2 (±9.6)§§§	36.0 (±9.3)§§§	29.6 (±9.0)
NY-Rest of State	94.8 (±3.1)	78.1 (±5.9)	89.8 (±3.8)	83.6 (±4.4)	60.1 (±8.5)	55.3 (±8.7)	45.6 (±8.9)	33.8 (±7.9)§§§	25.1 (±7.5)	12.5 (±5.9)
**HHS Region III**	**92.6 (±2.0)**	**80.4 (±3.4)**	**85.8 (±2.7)**	**79.7 (±3.2)**	**55.1 (±5.5)**	**48.0 (±5.4)**	**37.8 (±5.1)**	**36.5 (±5.0)** §§§	**24.5 (±4.5)** §§§	**14.6 (±3.7)** §§§
Delaware	95.3 (±2.4)	79.8 (±6.3)	84.4 (±4.6)	81.8 (±5.1)	68.7 (±8.1)	59.4 (±8.7)	51.7 (±8.9)	37.1 (±8.5)	25.0 (±7.5)	18.1 (±6.8)
Dist. of Columbia	85.9 (±8.6)	82.1 (±10.2)	83.1 (±8.3)	91.3 (±7.0)	55.6 (±14.6)	43.0 (±14.4)	30.2 (±12.3)	67.7 (±13.9)§§§	40.2 (±14.5)§§§	24.5 (±13.0)§§§
Maryland	93.8 (±3.9)	78.9 (±7.1)	83.2 (±6.2)	78.0 (±6.6)	50.0 (±11.5)	45.5 (±11.4)	33.4 (±10.7)	34.2 (±10.2)§§§	23.1 (±9.0)	NA
Pennsylvania	93.8 (±2.8)	92.1 (±3.4)	89.9 (±3.5)	90.4 (±3.6)	59.5 (±8.1)	53.5 (±8.2)	45.9 (±8.1)	44.1 (±7.8)§§§	26.8 (±6.9)§§§	15.4 (±5.5)§§§
PA-Philadelphia	90.2 (±4.6)	91.8 (±4.5)	89.6 (±4.1)	92.1 (±3.8)	78.4 (±7.3)	71.2 (±8.0)	54.5 (±9.1)	55.8 (±9.7)	35.7 (±8.9)	15.8 (±6.2)
PA-Rest of State	94.3 (±3.1)	92.2 (±3.8)	89.9 (±3.9)	90.2 (±4.0)	57.0 (±9.0)	51.1 (±9.1)	44.7 (±9.1)	42.7 (±8.7)§§§	25.7 (±7.7)§§§	15.4 (±6.2)
Virginia	92.0 (±4.9)	68.0 (±9.1)	83.6 (±6.5)	64.2 (±8.5)	51.9 (±12.7)	41.4 (±12.3)	27.6 (±10.6)	26.4 (±10.6)§§§	22.4 (±10.4)	NA
West Virginia	83.2 (±4.8)	59.4 (±8.1)	76.7 (±5.6)	77.3 (±5.5)§§§	49.7 (±9.4)	43.6 (±9.2)	38.4 (±9.0)	29.4 (±8.5)	19.2 (±7.3)	15.1 (±6.6)
**HHS Region IV**	**92.2 (±1.6)** ¶¶¶	**76.5 (±2.8)** §§§	**82.5 (±2.3)**	**70.9 (±2.6)** §§§	**53.0 (±4.1)** §§§	**42.9 (±4.1)**	**33.9 (±3.9)**	**28.4 (±3.8)** §§§	**18.6 (±3.2)** §§§	**11.1 (±2.6)** §§§
Alabama	93.4 (±3.2)	79.1 (±6.1)§§§	87.3 (±4.5)	69.5 (±6.0)	54.7 (±9.2)	46.6 (±9.2)	39.6 (±9.0)	18.4 (±6.9)	10.9 (±5.2)	NA
Florida	93.5 (±3.8)	76.0 (±7.0)	84.8 (±5.4)	72.3 (±6.4)	49.7 (±10.2)	40.7 (±10.0)	34.3 (±9.8)	27.8 (±8.6)	16.0 (±6.6)	13.2 (±6.2)
Georgia	96.4 (±3.1)	93.7 (±5.2)	82.0 (±6.6)	76.9 (±7.0)	53.7 (±10.8)	42.3 (±10.4)	33.2 (±9.5)	40.5 (±11.5)§§§	31.0 (±10.7)§§§	15.3 (±8.2)
Kentucky	92.7 (±3.7)	66.5 (±7.7)	84.4 (±5.1)	71.2 (±6.3)	47.6 (±9.8)	38.6 (±9.5)	26.8 (±8.5)	19.0 (±7.4)	10.8 (±5.2)	NA
Mississippi	92.3 (±3.9)	55.7 (±8.2)	60.2 (±6.7)	50.1 (±6.9)	53.1 (±9.5)	35.6 (±9.3)§§§	25.2 (±8.6)§§§	13.6 (±6.6)	NA	NA
North Carolina	87.1 (±4.7)¶¶¶	74.0 (±6.6)	89.4 (±4.0)	72.4 (±5.7)	59.3 (±9.5)	47.4 (±9.7)	32.8 (±9.1)	33.2 (±8.8)§§§	24.4 (±8.0)§§§	12.4 (±6.3)
South Carolina	91.0 (±3.8)	58.6 (±8.1)	71.9 (±6.6)	68.7 (±6.6)§§§	60.4 (±9.7)§§§	53.0 (±10.1)§§§	40.7 (±10.4)§§§	22.2 (±9.0)	13.1 (±6.7)	NA
Tennessee	88.4 (±4.6)	79.7 (±6.5)	80.0 (±5.4)	67.8 (±6.1)	48.9 (±9.5)	39.8 (±9.4)	35.9 (±9.1)	28.9 (±8.2)	18.0 (±7.1)	NA
**HHS Region V**	**93.6 (±1.3)** §§§	**83.0 (±2.4)** §§§	**86.3 (±1.9)** §§§	**79.9 (±2.1)** §§§	**57.4 (±3.8)** §§§	**46.1 (±3.8)** §§§	**35.0 (±3.6)**	**28.3 (±3.4)** †††	**18.2 (±2.9)** †††	**12.2 (±2.5)** †††
Illinois	93.5 (±2.5)§§§	79.9 (±5.1)§§§	86.2 (±4.2)§§§	79.0 (±4.5)§§§	53.2 (±7.6)§§§	42.6 (±7.5)§§§	33.8 (±7.2)§§§	34.8 (±7.5)	21.2 (±6.6)	16.5 (±6.4)
IL-City of Chicago	88.8 (±5.4)	79.4 (±8.3)	89.7 (±5.2)§§§	83.3 (±6.3)	61.8 (±12.7)	49.2 (±12.8)	38.6 (±12.1)	50.0 (±11.7)	29.1 (±10.4)	19.8 (±8.5)
IL-Rest of State	94.6 (±2.8)§§§	80.0 (±6.1)§§§	85.4 (±5.0)§§§	78.0 (±5.4)§§§	51.2 (±9.0)§§§	41.1 (±8.9)§§§	32.6 (±8.5)§§§	31.4 (±8.9)	19.4 (±7.8)	15.8 (±7.6)
Indiana	93.7 (±3.1)	91.8 (±4.2)	90.6 (±3.3)	93.5 (±2.7)	54.1 (±8.3)	44.2 (±8.2)	34.6 (±7.7)	18.2 (±6.3)	13.5 (±5.6)	8.1 (±4.3)
Michigan	94.0 (±3.4)	92.2 (±3.8)	81.0 (±5.2)	90.7 (±3.9)	66.0 (±9.1)§§§	49.4 (±9.9)	34.5 (±9.4)	30.0 (±8.1)§§§	16.8 (±6.7)	7.7 (±4.5)
Minnesota	94.0 (±3.2)	86.3 (±6.0)	91.4 (±3.8)	66.3 (±6.2)	59.3 (±9.3)	45.8 (±9.4)	37.6 (±9.0)	22.0 (±6.7)	13.1 (±5.6)	8.6 (±4.5)
Ohio	92.9 (±3.4)	66.2 (±7.4)	84.4 (±4.9)§§§	69.2 (±6.1)	54.8 (±9.3)	47.6 (±9.4)	35.0 (±8.8)	26.5 (±8.2)§§§	19.7 (±7.1)§§§	14.7 (±6.4)
Wisconsin	93.9 (±3.2)	93.4 (±3.7)	89.6 (±4.2)	81.4 (±4.9)	59.4 (±9.1)	47.8 (±9.4)	36.8 (±9.0)	31.7 (±8.5)§§§	20.9 (±7.7)§§§	13.7 (±6.7)
**HHS Region VI**	**89.0 (±2.5)**	**77.8 (±3.5)**	**84.9 (±2.6)**	**81.5 (±2.5)**	**56.2 (±5.2)**	**46.9 (±5.3)**	**38.1 (±5.2)** §§§	**33.1 (±4.6)** §§§	**24.2 (±4.2)** §§§	**14.5 (±3.3)** §§§
Arkansas	89.5 (±3.8)	59.6 (±7.6)	77.7 (±5.3)§§§	40.4 (±6.5)	44.3 (±9.3)	35.4 (±8.9)	24.4 (±8.0)	17.7 (±7.5)	NA	NA
Louisiana	97.4 (±2.4)§§§	89.1 (±4.4)	87.9 (±4.5)	87.7 (±4.4)	59.8 (±9.2)	54.1 (±9.6)	42.1 (±9.8)	27.0 (±8.1)	20.5 (±7.6)	13.5 (±6.6)
New Mexico	92.3 (±3.1)	72.5 (±6.3)§§§	85.6 (±4.5)	70.9 (±5.6)§§§	67.1 (±8.6)§§§	56.1 (±9.2)§§§	44.3 (±9.2)§§§	31.4 (±7.6)§§§	27.0 (±7.2)§§§	19.2 (±6.6)
Oklahoma	89.5 (±3.4)	67.3 (±6.2)	78.1 (±4.9)	66.2 (±5.4)	54.8 (±8.7)	46.5 (±8.7)	35.4 (±8.3)	45.2 (±7.5)§§§	31.1 (±6.9)§§§	17.3 (±5.7)
Texas	87.3 (±3.6)	79.4 (±4.9)	86.1 (±3.6)	87.6 (±3.5)	56.2 (±7.4)	46.3 (±7.4)	38.9 (±7.4)	34.1 (±6.5)§§§	25.2 (±5.9)§§§	15.0 (±4.6)§§§
TX-Bexar County	87.0 (±4.4)	78.9 (±6.2)	86.6 (±4.5)	87.2 (±4.2)	54.8 (±9.1)	45.7 (±9.2)	32.5 (±8.8)	32.4 (±8.7)§§§	19.1 (±6.9)	9.6 (±4.7)
TX-City of Houston	86.8 (±5.3)	82.1 (±7.2)	86.5 (±5.6)	91.4 (±4.6)	62.0 (±10.8)	51.9 (±11.3)	33.9 (±10.6)	40.3 (±9.8)	27.8 (±8.5)	17.5 (±7.0)
TX-Rest of State	87.3 (±4.1)	79.2 (±5.6)	86.0 (±4.1)	87.4 (±4.0)	55.9 (±8.6)	45.9 (±8.6)	39.8 (±8.5)	33.7 (±7.4)§§§	25.4 (±6.8)§§§	15.2 (±5.3)§§§
**HHS Region VII**	**89.0 (±2.5)**	**67.4 (±4.5)**	**82.4 (±2.9)**	**62.5 (±3.7)**	**49.7 (±5.6)**	**41.7 (±5.4)**	**31.7 (±4.9)**	**26.0 (±4.4)** §§§	**16.6 (±3.5)** §§§	**9.4 (±2.7)** §§§
Iowa	90.2 (±4.0)	62.1 (±7.4)	79.6 (±5.0)	63.6 (±5.9)	57.0 (±8.7)	52.2 (±8.8)	41.9 (±8.8)	30.3 (±8.0)	24.0 (±7.3)§§§	13.7 (±5.4)
Kansas	86.9 (±4.6)	80.7 (±6.1)	84.6 (±4.9)¶¶¶	55.9 (±6.8)	39.9 (±9.9)	29.9 (±9.2)	21.0 (±8.2)	25.1 (±8.6)§§§	19.3 (±7.8)	NA
Missouri	88.3 (±4.8)	58.6 (±8.6)	81.5 (±5.4)	60.7 (±7.1)	46.1 (±10.6)	38.1 (±10.1)	28.8 (±9.0)	20.5 (±7.7)	NA	NA
Nebraska	92.3 (±3.2)	84.6 (±5.5)	86.1 (±4.7)	77.5 (±5.2)	65.1 (±9.2)	55.3 (±9.3)	41.5 (±9.1)	38.2 (±8.7)§§§	26.4 (±7.8)§§§	19.7 (±7.2)§§§
**HHS Region VIII**	**91.0 (±2.1)**	**71.8 (±3.8)**	**86.1 (±2.5)**	**67.0 (±3.3)**	**52.6 (±5.0)**	**43.4 (±5.0)**	**33.1 (±4.7)**	**24.3 (±4.4)**	**16.6 (±3.8)**	**8.6 (±2.5)**
Colorado	92.4 (±3.3)	78.5 (±5.7)	87.1 (±4.4)¶¶¶	73.6 (±5.6)	58.2 (±8.6)	50.0 (±8.8)	39.1 (±8.7)	33.5 (±8.6)	21.7 (±7.5)	9.9 (±4.8)
Montana	90.5 (±4.0)	58.6 (±8.6)	84.3 (±5.1)	51.6 (±6.6)	45.8 (±9.6)	37.9 (±9.0)	28.3 (±8.1)¶¶¶	23.8 (±8.1)	17.2 (±7.0)	9.4 (±4.6)
North Dakota	96.1 (±1.9)§§§	86.0 (±5.3)§§§	95.0 (±2.9)	93.7 (±3.2)	57.5 (±9.4)	51.0 (±9.4)	41.1 (±9.1)	36.1 (±9.1)§§§	26.6 (±8.4)§§§	18.4 (±7.5)
South Dakota	94.1 (±3.2)	50.6 (±8.4)	70.0 (±6.4)	51.7 (±6.7)§§§	56.0 (±9.7)	52.0 (±9.7)	42.3 (±9.6)	22.1 (±7.1)	17.0 (±6.4)	8.4 (±4.2)
Utah	87.5 (±4.6)	62.2 (±8.4)	86.2 (±4.9)	61.0 (±6.7)	44.3 (±9.6)	30.9 (±8.9)	20.5 (±7.8)	11.0 (±5.8)	NA	NA
Wyoming	90.6 (±4.0)	90.1 (±4.5)	92.3 (±3.0)§§§	63.1 (±6.2)	54.3 (±9.4)	49.5 (±9.4)	42.1 (±9.3)	16.6 (±6.0)	12.3 (±5.3)	8.4 (±4.5)
**HHS Region IX**	**90.7 (±3.4)**	**77.4 (±5.2)**	**89.7 (±3.5)**	**80.6 (±4.5)**	**66.0 (±7.5)**	**54.8 (±8.0)**	**43.3 (±8.1)**	**48.7 (±7.8)** §§§	**32.5 (±7.7)** §§§	**16.4 (±6.3)**
Arizona	85.4 (±4.6)	67.8 (±7.1)	84.4 (±5.0)	86.7 (±4.6)	64.1 (±8.7)	47.9 (±9.5)	37.4 (±9.2)	44.4 (±8.7)§§§	33.5 (±8.4)§§§	19.5 (±6.9)
California	91.5 (±4.2)	79.0 (±6.5)	91.1 (±4.4)	80.9 (±5.7)	67.6 (±9.4)	57.3 (±10.0)	45.8 (±10.2)	50.9 (±9.7)§§§	33.2 (±9.7)§§§	16.6 (±8.0)
Hawaii	90.4 (±4.5)	83.3 (±5.7)	80.2 (±5.4)	75.0 (±6.0)	52.7 (±10.1)	46.6 (±10.0)	34.4 (±9.5)	39.7 (±8.9)	29.0 (±8.1)	15.1 (±6.0)
Nevada	92.8 (±3.5)	74.6 (±6.6)	88.3 (±4.1)	64.0 (±6.1)	53.8 (±9.4)	38.9 (±9.2)	27.4 (±8.3)	31.9 (±8.5)§§§	20.4 (±7.2)	7.3 (±3.9)
**HHS Region X**	**90.0 (±2.4)**	**75.3 (±4.6)**	**84.1 (±3.0)**	**72.7 (±3.5)** §§§	**61.0 (±5.8)**	**51.2 (±5.9)**	**40.7 (±5.9)**	**32.0 (±5.0)** §§§	**19.3 (±4.0)** §§§	**11.6 (±3.2)** §§§
Alaska	92.0 (±3.7)	80.7 (±6.1)	74.3 (±5.8)	55.2 (±6.5)	52.2 (±9.4)	36.1 (±9.0)	27.1 (±8.2)	27.6 (±7.9)§§§	17.8 (±6.9)§§§	8.5 (±4.7)
Idaho	85.2 (±5.5)	63.8 (±9.4)	74.6 (±6.6)§§§	71.6 (±7.0)	55.0 (±10.6)	45.8 (±10.5)	31.3 (±9.6)	34.5 (±10.2)§§§	21.6 (±8.8)	NA
Oregon	92.3 (±3.2)	84.3 (±4.7)§§§	87.0 (±4.3)	65.3 (±5.8)	66.3 (±8.4)	54.9 (±8.8)	39.5 (±8.8)	35.8 (±8.1)§§§	20.8 (±6.9)§§§	12.2 (±5.0)
Washington	89.9 (±4.0)	71.6 (±8.1)	86.2 (±5.0)	79.0 (±5.6)	60.7 (±9.7)	52.3 (±9.9)	45.3 (±9.8)	29.8 (±8.0)§§§	18.0 (±6.3)§§§	12.5 (±5.2)
***Range***	***(83.2–97.4)***	***(50.6–95.8)***	***(60.2–95.5)***	***(40.4–93.7)***	***(39.9–76.6)***	***(29.9–68.5)***	***(20.5–56.5)***	***(11.0–69.3)***	***(10.8–58.0)***	***(7.3–43.2)***
**Territory**										
Guam	84.8 (±4.6)	43.7 (±8.5)	73.8 (±5.4)	72.4 (±5.7)	69.1 (±8.2)	45.2 (±8.9)	33.6 (±8.3)	21.8 (±7.0)	8.6 (±4.2)	NA
U.S. Virgin Islands	92.0 (±3.0)	77.9 (±5.3)	76.4 (±5.2)	38.4 (±6.0)	33.2 (±8.5)	17.7 (±6.7)	9.5 (±4.9)	17.2 (±6.6)	NA	NA

**Abbreviations:** CI = confidence interval; HHS = U.S. Department of Health and Human Services; MMR = measles, mumps, and rubella; VAR = varicella; Tdap = tetanus toxoid, reduced diphtheria toxoid, and acellular pertussis; MenACWY = meningococcal conjugate; HPV = human papillomavirus; NA = not available (estimate not reported because unweighted sample size for the denominator was <30 or 95% CI half width/estimate >0.6).

* Vaccination estimates for additional measures, including ≥3 doses hepatitis B, and ≥1 dose varicella vaccines are available at http://www.cdc.gov/vaccines/stats-surv/nis/default.htm#nisteen.

† Adolescents (N = 18,264) in the 2013 NIS-Teen were born January 11, 1995, through February 13, 2001.

§ ≥2 doses of MMR vaccine.

¶ ≥2 doses of VAR vaccine among adolescents without a reported history of varicella.

** ≥1 dose Tdap vaccine on or after age 10 years.

†† ≥1 dose of MenACWY or meningococcal-unknown type vaccine.

§§ ≥1 dose of HPV vaccine, either quadrivalent or bivalent may be used for females, and only quadrivalent may be used for males. For ≥1, ≥2, and ≥3 dose measures, separate percentages are reported among females only (n = 8,710) and among males only (n = 9,554).

¶¶ ≥2 doses of HPV vaccine, either quadrivalent or bivalent may be used for females, and only quadrivalent may be used for males.

*** ≥3 doses of HPV vaccine, either quadrivalent or bivalent may be used for females, and only quadrivalent may be used for males.

††† Estimates with 95% CI half-widths >10 might not be reliable.

§§§ Statistically significant (p<0.05) percentage point increase from 2012.

¶¶¶ Statistically significant (p<0.05) percentage point decrease from 2012.

## Footnotes

* Adolescents who receive their first MenACWY vaccine dose as routinely recommended at age 11–12 years should receive a second dose at 16 years. Adolescents who receive their first dose at ages 13–15 years should receive a second dose at age 16–18 years, with a minimum interval of ≥8 weeks between doses. Adolescents who receive a MenACWY vaccine dose at age ≥16 years do not need a second dose.

† Catch-up vaccination is recommended for some vaccines routinely recommended in childhood. MMR vaccine is recommended for any adolescents who have not had 2 doses of MMR vaccine. Catch-up vaccination for varicella is recommended for persons aged 7–18 years without evidence of immunity (MMWR 2007;56 [No. RR-4]). Adolescents should have received 2 doses of varicella vaccine. Catch up vaccination for hepatitis B is recommended for any unvaccinated persons, and they should complete a 3-dose series. However, a 2-dose series (doses separated by at least 4 months) of adult formulation Recombivax HB is licensed for use in children aged 11–15 years (1).

§ Eligible participants were born during January 1995–February 2001. Except as noted, coverage for ≥1 and ≥2 varicella doses were obtained among persons with no history of varicella disease. HPV vaccination coverage represents receipt of any HPV vaccine and does not distinguish between bivalent or quadrivalent vaccines. Some adolescents, both males and females, might have received more than the 3 recommended HPV doses. Influenza vaccination coverage estimates are not included in this report but are available online at http://www.cdc.gov/flu/fluvaxview/index.htm.

¶ *HP2020* objectives and targets were established for females only in 2010 (4), before ACIP’s 2011 recommendation for routine use of the quadrivalent HPV vaccine among males aged 11–12 years (2). In April 2014, the federal *HP2020* work group approved a new *HP2020* objective to reach an 80% vaccination target with 3 doses of HPV vaccine among adolescent males aged 13–15 years (Office of Policy, Office of the Director, National Center for Immunization and Respiratory Diseases, CDC, unpublished data, April 2014).

** Local areas that received Federal Section 317 immunization funds were sampled separately: Chicago, Illinois; New York, New York; Philadelphia County, Pennsylvania; Bexar County, Texas; and Houston, Texas.

†† All identified cell phone households were eligible for interview. Sampling weights adjusted to correct for dual-frame (landline and cell phone) sampling, nonresponse, noncoverage, and overlapping samples of mixed telephone users. A description of NIS-Teen dual-frame survey methodology and its effect on reported vaccination estimates is available at http://www.cdc.gov/vaccines/stats-surv/nis/dual-frame-sampling-082812.htm.

§§ The Council of American Survey Research Organizations (CASRO) response rate for the landline and cell phone samples were 51.1% and 23.3%, respectively. For completed interviews, 6,039 by landline (59.5%) and 12,225 by cell phone (54.5%) had adequate provider data. Overall, 33% of completed interviews with adequate provider data were from landlines, and 67% were from cell phones. For USVI, the landline and cell phone sample CASRO rate was 60.6% and 31.5%, respectively. For Guam, landline and cell phone sample CASRO was 45.6% and 21.0%, respectively. The CASRO response rate is the product of three other rates: 1) the resolution rate (the proportion of telephone numbers that can be identified as either for business or residence), 2) the screening rate (the proportion of qualified households that complete the screening process), and 3) the cooperation rate (the proportion of contacted eligible households for which a completed interview is obtained).

¶¶ Adolescents from the U.S. Virgin Islands (156 females and 176 males) and from Guam (164 females and 199 males) were excluded from the national estimates.

*** Additional information available at ftp://ftp.cdc.gov/pub/health_statistics/nchs/dataset_documentation/nis/nisteenpuf12_dug.pdf.

††† Annual estimates of vaccination coverage were regressed on survey year via a weighted linear regression, with regression weights calculated using the inverse of the estimated variance of the vaccination coverage point estimate.

§§§ The completion rate for 3-dose HPV vaccination series represents the percentage of adolescents who received ≥3 doses among those who had ≥1 HPV dose and ≥24 weeks between the first dose and the interview date.

¶¶¶ Adolescents were classified as below the federal poverty level if their family’s total income was less than the federal poverty level specified for their family size and number of children aged <18 years. All others were classified as at or above the poverty level. Poverty status was unknown for 432 adolescents. Additional information available at http://www.census.gov/hhs/www/poverty.

**** Children aged ≤18 years who are Medicaid-eligible, uninsured, or American Indian/Alaska Native (as defined by the Indian Health Care Improvement Act) are eligible to receive vaccines from providers through the VFC program. Children categorized as “underinsured” (because their health plans do not include coverage for recommended vaccinations) are eligible to receive VFC vaccines if they are served by a rural health clinic or federally qualified health center or under an approved deputization agreement. Additional information is available at http://www.cdc.gov/vaccines/programs/vfc/providers/eligibility.html.

†††† Additional information available at http://www.amstat.org/meetings/jsm/2012/onlineprogram/abstractdetails.cfm?abstractid=304324.

## References

[1] Akinsanya-Beysolow I, Advisory Committee on Immunization Practices (ACIP), ACIP Child/Adolescent Immunization Work Group (2014). Advisory Committee on Immunization Practices recommended immunization schedules for persons aged 0 through 18 years—United States, 2014. MMWR.

[2] CDC (2011). Recommendations on the use of quadrivalent human papillomavirus vaccine in males—Advisory Committee on Immunization Practices (ACIP), 2011. MMWR.

[3] CDC (2011). General recommendations on immunization: recommendations of the Advisory Committee on Immunization Practices (ACIP). MMWR.

[4] US Department of Health and Human Services (2012). Healthy People 2020.

[5] Community Preventive Services Task Force (2014). Increasing appropriate vaccination.

[6] CDC (2011). Updated recommendations for use of meningococcal conjugate vaccines—Advisory Committee on Immunization Practices (ACIP), 2010. MMWR.

[7] CDC (2013). National and state vaccination coverage among adolescents aged 13–17 years—United States, 2012. MMWR.

[8] Holmon DM, Bernard V, Roland KB, Watson M, Liddon N, Stokley S (2014). Barriers to human papillomavirus vaccination among U.S. adolescents: a systematic review of the literature. JAMA Pediatr.

[9] Stokley S, Jeyarajah J, Yankey D (2014). Human papillomavirus vaccination coverage among adolescents, 2007–2013, and postlicensure vaccine safety monitoring, 2006–2014—United States. MMWR.

[10] CDC (2013). National, state, and local area vaccination coverage among children aged 19–35 months—United States, 2012. MMWR.

